# Changes in T-cell subpopulations and cytokine network during early period of ibrutinib therapy in chronic lymphocytic leukemia patients: the significant decrease in T regulatory cells number

**DOI:** 10.18632/oncotarget.16148

**Published:** 2017-03-13

**Authors:** Monika Podhorecka, Aneta Goracy, Agnieszka Szymczyk, Malgorzata Kowal, Blanca Ibanez, Olga Jankowska-Lecka, Arkadiusz Macheta, Aleksandra Nowaczynska, Elzbieta Drab-Urbanek, Sylwia Chocholska, Dariusz Jawniak, Marek Hus

**Affiliations:** ^1^ Department of Haematooncology and Bone Marrow Transplantation, Medical University of Lublin, Lublin, Poland

**Keywords:** chronic lymphocytic leukemia, cytokines, ibrutinib, T-cells, T regulatory cells

## Abstract

B cell receptor (BCR) stimulation signal plays an important role in the pathogenesis of chronic lymphocytic leukemia (CLL), and kinase inhibitors directed toward the BCR pathway are now the promising anti-leukemic drugs. Ibrutinib, a Bruton tyrosine kinase inhibitor, demonstrates promising clinical activity in CLL. It is reported that ibrutinib, additionally to directly targeting leukemic cells, also inhibits the interactions of these cells with T cells, macrophages and accessory cells. Assessment of these mechanisms is important because of their non -direct anti-leukemic effects and to identify possible side effects connected with long-term drug administration.

The aim of this study was to assess the *in vivo* effects of ibrutinib on T-cell subpopulations and cytokine network in CLL. The analysis was performed on a group of 19 patients during first month of ibrutinib therapy. The standard multicolor flow cytometry and cytometric bead array methods were used for assessment of T-cell subsets and cytokines/chemokines, respectively.

The data obtained indicates that Ibrutinib treatment results in changes in T-cell subpopulations and cytokine network in CLL patients. Particularly, a significant reduction of T regulatory cells in peripheral blood was observed. By targeting these populations of T cells Ibrutinib can stimulate rejection of tumor cells by the immune system.

## INTRODUCTION

Chronic lymphocytic leukemia (CLL), the most frequent type of adult leukemias in Western Countries, belongs to the group of lymphoproliferative disorders. It is defined as the disease of accumulation of mature monoclonal B cells and not their proliferation resulted from a defective apoptotic process [1, 2, 3]. The significant heterogeneity in the course of the disease between CLL patients is reported. A number of prognostic parameters like mutation status of immunoglobulin variable heavy chain (IgVH) genes, the expression of ZAP-70 or CD38 on leukemic cells, or the presence of genetic abnormalities may predict favorable or poor prognosis among leukemic patients and distinguish those who could develop aggressive disease and need immediate treatment [4, 5]. Among prognostic factors, the del17p or mutation of the *TP53* gene, are associated with a worse prognosis [6, 7]. These mutations are the cause of resistance to most chemotherapeutic agents used in the treatment of CLL because they mediate p53-dependent apoptosis [8, 9].

Recently, a great progress has been made in therapy of CLL. Present treatment options involve a combination of conventional chemotherapeutics, monoclonal antibodies and targeted signaling inhibitors. The combination of fludarabine, cyclophosphamide and rituximab, is the conventional first-line of treatment for patients without relevant co-existing disorders, who do not display the high-risk genetic features [6]. The elderly or non-fit patients, should receive bendamustine or chlorambucil with an anti-CD20 antibody [6]. In 2014, two novel agents, blocking the BCR signaling pathway, idelalisib and ibrutinib, were approved as first-line treatment for patients with poor prognostic parameters and for the relapsed disease [10, 11]. Idelalisib targets phosphatidylinositol-3-kinase (PI3K), while ibrutinib is a Bruton's tyrosine kinase (BTK) inhibitor. These drugs interrupt BCR signaling leading to the reduction of leukemic cells number. The direct effects of ibrutinib on CLL cells are clearly observed; however, its influence on the accessory cells, particularly *in vivo*, is less well defined. The aim of this study was to assess the *in vivo* effects of ibrutinib on T-cell subpopulations and cytokine network in CLL. The analysis was performed in a group of 19 patients during first month of ibrutinib therapy.

## RESULTS

### Changes in main lymphocyte subsets during ibrutinib therapy

**Figure 1:**
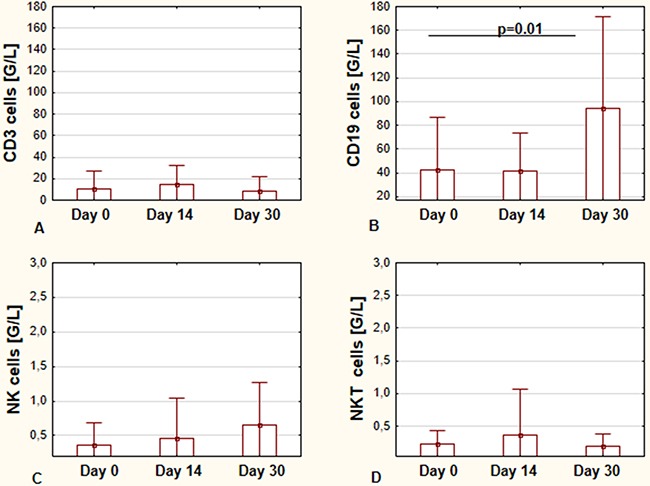
The effects of ibrutinib on the main lymphocyte subsets during the first month of therapy Total number of CD19+ cells before starting treatment (day 0), at day 14, and day 30, respectively **(A)** Total number of CD3+ cells at day 0, day 14, and day 30 of treatment, respectively **(B)** The number of NK cells at day 0, day 14, and day 30 of treatment, respectively **(C)** The number of NKT cells at day 0, day 14, and day 30 of treatment, respectively **(D)** All graphs show the mean ± standard deviation of results obtained from the group of analyzed patients (n=19). The p values are indicated.

Figure 1 shows the effect of ibrutinib on the main lymphocyte subsets during the first month of therapy. The changes in the number of CD19+, CD3+, NK (Natural killer), and NKT (Natural killer T) lymphocytes were assessed. In the analyzed period, we observed significant differences in numbers of CD19+ cells from day 0 to day 30 - the mean values at day 30 were higher in comparison to those on day 0 (Figure 1A). Total number of CD3+ cells was lower on day 30 of therapy in comparison to day 0; however, the difference was not statistically significant (Figure 1B). The increase in NK cell count was observed; however, also without statistical significance. Lastly, NKT cells number remained at comparable level. Values for NK and NKT cells are shown in Figure 1C and 1D, respectively.

### Changes in naive and memory T-cells during ibrutinib therapy

**Figure 2:**
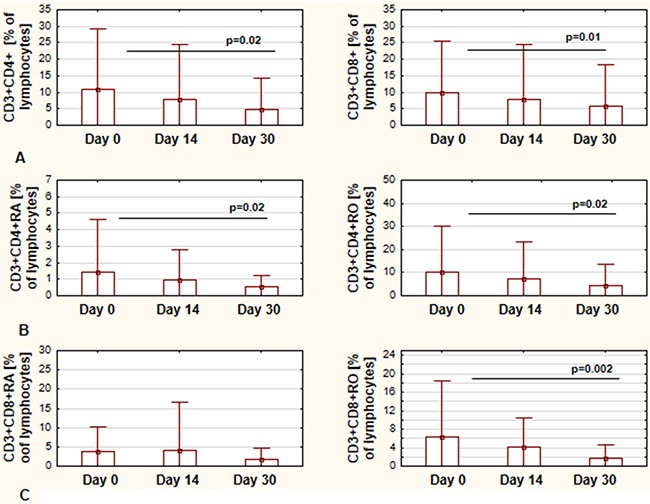
Changes in CD4+ and CD8+ T-cells during ibrutinib therapy The percentage of CD4+CD3+ cells and CD8+CD3+ calculated in regards to lymphocytes population at day 0, day 14, and day 30 of treatment, respectively **(A)** The percentage of CD4RA and CD4RO cells calculated in regards to lymphocytes population at day 0, day 14, and day 30 of treatment, respectively **(B)** The percentage of CD8RA and CD8RO cells calculated in regards to lymphocytes population at day 0, day 14, and day 30 of treatment, respectively **(C)** All graphs show the mean ± standard deviation of results obtained from the group of analyzed patients (n=19). The p values are indicated.

The next step of the study was to assess the CD4 and CD8 populations of T cells. There were no statistically significant differences in the number of CD4 and CD8 cells during first month of ibrutinib therapy. The CD4/CD8 ratio did not change, neither. However, we observed significant lower percentages for both, CD4+CD3+ and CD8+CD3+ cells, in regards to lymphocyte population (Figure 2A). Among CD4+CD3+ cells, both CD4RA and CD4RO representing the naïve and memory cells, respectively, were significantly decreased in the first month of therapy (Figure 2B). In CD8+CD3+ population only the percentage of CD8RO cells was decreased, while there was no difference in percentage of CD8RA cells (Figure 2C).

### Influence of ibrutinib therapy on Treg cells

**Figure 3:**
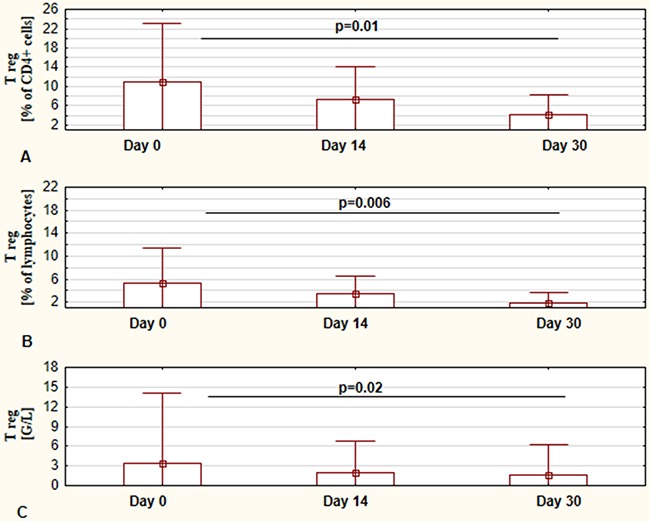
Influence of ibrutinib therapy on Treg cells (CD4+CD25^high^FoxP3+ crlls) The percentage of CD4+CD25^high^FoxP3+ T cells calculated in regards to lymphocytes population at day 0, day 14, and day 30 of treatment, respectively **(A)** The percentage of CD4+CD25^high^FoxP3+ T cells calculated in regards to CD4+ lymphocytes population at day 0, day 14, and day 30 of treatment, respectively **(B)**The number of CD4+CD25^high^FoxP3+ T cells at day 0, day 14, and day 30 of treatment, respectively **(C)** The presented graphs show the mean ± standard deviation of results obtained from the group of analyzed patients (n=19). The p values are indicated.

**Figure 4:**
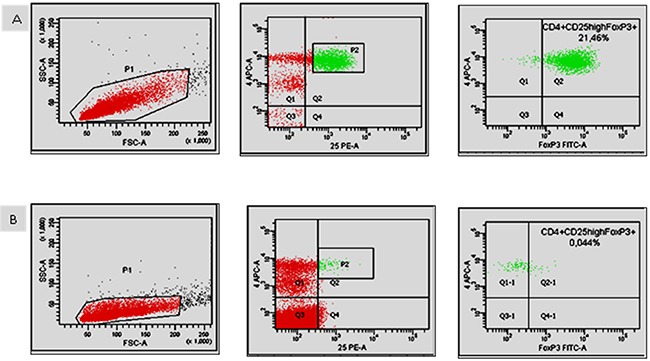
Representative flow cytometric dot plots showing the percentage of Treg cells before commencing therapy (A) and after 30 days of treatment (B) The assessment was performed in two steps using a sequential gating strategy. Tregs are identified as CD4+CD25^high^FoxP3+ T cells.

In the presented study the assessment of regulatory FOXP3+ cells was also performed. The results of changes in the number and percentage of these cells at days 0, 14, and 30 of ibrutinib therapy are shown in Figure 3. Interestingly, we observed a significantly lower number of FOXP3+ T cells at 30 day in comparison to values before commencing treatment (Figure 3A). Similarly, the percentage of Tregs was statistically significant decreased in course of ibrutinib therapy (Figure 3B and 3C). Representative flow cytometry dot plots illustrating the differences in number of Treg cells between day 0 and day 30 are presented in Figure 4.

### Ibrutinib-induced changes in the cytokines and chemokines level

In course of ibrutinib therapy statistically significant differences were observed only in regard to IL-10 level. The concentration of this cytokine was statistically significant lower at day 14 and at day 30 in comparison to pre-treatment concentration (day 0). The differences in regard to other cytokines analyzed in the study were not statistically significant. Analysis of chemokines levels during ibrutinib therapy showed statistically significant decrease in IP-10, MCP and IL-8 levels. RANTES concentration was higher at day 14 and 30 in comparison to day 1 of ibrutinib therapy, however the difference was not statistically significant.

### Comparison of ibrutinib effects in standard versus poor prognosis patients

To assess whether effect of ibrutinib therapy is connected with CLL prognostic factors, the data was compared in good versus poor prognosis cases. The ZAP-70 positive versus ZAP-70 negative groups, CD38 positive versus CD38 negative groups, early (0-2) versus advanced (3-4) stages of CLL according to the Rai classification and standard cytogenetic risk versus high cytogenetic risk cases were analyzed, respectively. We did not find statistically significant differences, thus prognostic factors seem not to influence ibrutinib effects.

## DISCUSSION

BCR stimulation signal launches a signaling cascade leading to survival of leukemic cells and also influences tissue homing and microenviroment [12, 13, 14]. Inhibition of BCR cascade can be mediated through different pathways, including spleen tyrosine kinase (Syk), BTK and PI3K [15]. BTK is a member of Tec family kinases and is activated upstream by Src-family kinases. BTK starts downstream activation of critical cell survival pathways, such as nuclear factor κB (NFκB) and mitogen activated protein-kinase (MAPK) [13, 16]. BTK mutation in humans results in X-linked agammaglobulinemia [13, 17].

The central role of BCR in disease pathogenesis is that of introducing kinase inhibitors directed toward the BCR pathway as a new promising anti-leukemic therapy [18, 19]. Ibrutinib, a first-in-class, orally administered inhibitor of BTK, has demonstrated promising pre-clinical and clinical effects in CLL [20]. The clinical studies in relapsed/refractory older patients with CLL reported high response rates and tolerable toxicity of the drug [20, 21, 22]. The phase 3 clinical trail in patients with previously treated CLL demonstrated a statistically significant reduction in the rate of progression and death in comparison to ofatumumab therapy [23]. Based on these results, single-agent ibrutinib was approved for previously treated CLL patients and for all patients with del(17p) [20] The prolonged follow-up studies confirmed that ibrutinib therapy leads to long-lasting remissions. In contrast to chemotherapy, which is given for a defined time, the side-effects of ibrutinib are moderate, allowing to continue the therapy for longer time to increase proportion of responses [20].

Leukemic cells of CLL depend on interactions with the microenvironment that are beneficial to their survival via direct cell contact or through secretion of cytokines. On the other hand, CLL cells produce cytokines that influence T cells, macrophages or accessory cells and inhibit anti-tumor activity [24, 25]. Disrupting these tumor-microenvironment interactions seems to be critical for progression of the disease. It was reported that BCR-directed kinase inhibitors, additionally to targeting leukemic cells, inhibit interactions of these cells with the cells of immune system. Numerous *in vitro* experiments have shown that ibrutinib influences T cells and cytokines network; however, less is known about such effects *in vivo*. Assessment of these mechanisms is important because of their non-direct anti-leukemic effects and to identify possible side effects connected with long-term drug administration. In the presented study we focused on evaluation of changes in lymphocytes, particularly T cells, as well as cytokines and chemokines in patients treated with ibrutinib during the first month of therapy. Recently, similar studies focusing on disruption of *in vivo* CLL tumor-microenvironment interactions by ibrutinib were published as a result of Phase II study of ibrutinib monotherapy [26, 27]. The authors indicate that serum levels of key chemokines and inflammatory cytokines decreased significantly in patients on ibrutinib. Moreover, therapy decreased the overall T-cell numbers, especially the Th17 subset of CD4 T cells. They also found that in the bone marrow microenvironment, the drug destroyed the interactions between macrophages and leukemic cells by blocking CXCL13 secretion and decreasing the chemoattraction of neoplastic cells [26]. Similarly to results of Niemann et al. [26], we observed a decreased in T-cell number and stable CD4/CD8 ratio. Moreover, we observed significant lowering of percentage of both CD4+CD3+ and CD8+CD3+ cells. Among CD4+CD3+ cells, both CD4RA and CD4RO naïve and memory cells, respectively, were significantly decreased. In CD8+CD3+ population, only the percentage of CD8RO cells was decreased, while there was no difference in percentage of CD8RA cells. Additionally we analyzed NK and NKT populations; however, the differences in total number of these cells were not statistically significant. We detected an increase in total number of CD19+ cells after first month of treatment. This is confirming results of treatment with ibrutinib described by Byrd et al. [20] where number of lymphocytes was the highest after 4 weeks of treatment. Such lymphocytosis was resolved with continued therapy after 19 weeks. Also Niemann et. al [26] reported reduction in CD19+ tumor cell number after longer therapy with ibrutinib up to 24 to 48 weeks.

In the presented study, we report for the first time that ibrutinib therapy reduced the number and percentage of T regulatory CD4+CD25^high^FoxP3+ cells. Treg cells play an important role in the control of immunosurveliance [28]. It was reported that the frequency of Tregs was significantly higher in CLL patients in comparison to healthy persons and it is higher in more advanced in comparison to early clinical stages [29]. These cells are able to block T cell response against leukemic cells. Thus, reducing the level of Treg cells may permit anti-leukemic action of T cells [29]. Ibrutinib therapy resulted in a significant reduction of T regulatory cells in peripheral blood shortly after beginning the therapy. Ibrutinib, by targeting these populations of T cells, can stimulate rejection of tumor cells by the immune system. Such effects on Tregs in CLL were also reported for other anti-leukemic drugs such as fludarabine or thalidomide [30, 31]. Moreover, effects of ibrutinib on Treg cells seem to be independent of expression of poor prognosis factors.

The last stage of our study was focused on an assessment of cytokine and chemokine concentration during ibrutinib therapy. Firstly, we analyzed cytokines secreted by T helper cells subsets: Th1/Th2/Th17. We observed significant lowering of IL-10 concentration, the cytokine produced by Th2 cells, however no differences in Th1 and Th17 cells. It was previously reported that ibrutinib alter the composition of T lymphocytes subpopulations by suppression of Th1 cells and inhibition of differentiation of Th17 T cells [26, 32]. The levels of most chemokines analyzed in our study decreased during first month of treatment, that is consistent with results of Niemann et al. [26]. We observed, however an growth in RANTES concentration. The increase in RANTES level was reported as result of BCR stimulation and it was reduced by BCR inhibitors [33]. Further studies, however, are required to elucidate the status of cytokines and chemokines in longer therapy period.

In conclusion, CLL cells depend on T cells and cytokines interactions for their survival that are partially mediated via BCR signals. The treatment with new BCR-inhibitors, like ibrutinib, leads to the reduction of tumor cells number both directly and via microenvironment signals. The presented study underscores the possible influence of ibrutinib on T cells subpopulations showing statistically significant decrease of Treg cell subset during first month of therapy. Further studies should elucidate precisely the role of the drug in T cell subsets nd cytokines modulation. It may be of importance for the effectiveness of treatment, as well as possible side effects of long-term therapy

## MATERIALS AND METHODS

### Clinical characteristics of analyzed patients

Nineteen CLL patients who were diagnosed in the Department of Hematooncology, Medical University of Lublin, and who were qualified for ibrutinib therapy, constituted the study group. The diagnosis of CLL was based on clinical examinations and morphological and immunological standards. All subjects were previously treated with other regimens. The clinical characteristics are shown in Table 1. The local Bioethics Committee granted permission to conduct the research and patients were asked to sign informed consents. Samples of peripheral blood for mononuclear cells and serum preparation were collected on days 0, 14, and 30 of ibrutinib therapy and then were subjected to further procedures.

**Table 1:** Clinical characteristics of the analyzed ibrutinib-treated CLL patients

Characteristics	Median (range)	Number of patients/ percentage
**Sex**		
Female		7
Male		12
**Age**		
**Diagnosis**	59 (43-67)	
**Ibrutinib therapy**	65 (47-75)	
**Rai stadium**		
0		0
I		0
II		9
III		2
IV		8
**CD38 expression**		
**Negative**		65 %
**Positive**		35 %
**ZAP-70 expression**		
Negative		55 %
Positive		45 %
**Cytogenetics**		
Standard-risk		25 %
High-risk		45 %
Not available		30%
**Number of previous therapy lines**	3 (1-7)	

### Mononuclear cells isolation and specimens storage

Peripheral blood mononuclear cells were separated via density gradient centrifugation in Biocoll separating solution (density: 1.077 g/mL, Biochrom, Germany) and washed with phosphate-buffered saline. Then the number and viability of cells were assessed with trypan blue staining. Viability below 95% was a disqualifying criterion for further study. Mononuclear cells were stored in liquid nitrogen until staining procedures. Serum was isolated and cryopreserved at –80°C. The samples were thawed at the time of analysis.

### Assessment of T cell subpopulations by flow cytometry method

The monoclonal antibodies directed against surface cell antigens were used to distinguish particular cell populations (Table 2). All monoclonal antibodies were purchased from Becton Dickinson, USA. They were conjugated to one of the four fluorochromes: fluorescein isothiocyanate (FITC) or alexa fluor 488 (AF488), respectively, phycoerithrin (PE), peridin chlorophyll protein (PE-Cy5), and allophycocyanin (APC). The cells and antibody mixture were incubated for 20 minutes at room temperature in the dark, then washed twice, suspended again in phosphate-buffered saline and subjected to acquisition.

**Table 2:** T-cell subpopulations assessed in the study with use of flow cytometry method

FITC or AF 488	PE	PE-Cy5	APC	T cell subpopulation
IgG1	IgG2a	IgG1	IgG1	negative control
CD45	CD14			lymphocytes, monocytes, granulocytes
CD3	CD16+CD56+	CD45	CD19	T, NK, B cells
CD45RA	CD45RO	CD3	CD4	resting and memory T helper cells
CD45RA	CD45RO	CD3	CD8	resting and memory T cytotoxic cells
FOXP3	CD25	CD4		regulatory T cells

Abbreviations: FITC, Fluorescein isothiocyanate; AF, Alexa Fluor; PE, Phycoerythrin; APC, allophycocyanin

The assessment of regulatory T cells (Tregs) was performed in two steps. First, monoclonal antibodies against CD4 PE-Cy5 and CD25 PE were used to stain surface markers. Next, fixation and permeabilization procedures were performed. Then, co-staining of intracellular FoxP3 via application of the AF488 conjugated anti-FoxP3 antibody was done. Using a sequential gating strategy Tregs were identified as CD4+CD25^high^FoxP3+ T cells.

Acquisition and analysis of the data were carried out by flow cytometry method using the eight-color flow cytometer, FACSCanto II with FACSDiva Software (Becton Dickinson, USA). At least 10,000 cells of each sample were collected. Cell populations were initially gated according to forward scatter and side scatter differentiation. For T lymphocyte analysis additional gates for CD3, CD4, or CD8 cells were established. Analysis was performed with the use of FACSDiva software. The results were reported as percentages of lymphocyte population. Based on percentages and lymphocyte concentration the absolute cell numbers were calculated.

### Measurement of cytokine and chemokine concentrations with CBA method

Assessment of cytokine and chemokine con-centrations in CLL patients during ibrutinib therapy was performed with use of cytometric bead array (CBA) method. Human Th1/Th2/Th17 Cytokine Kit and Human Chemokine Kit (Becton Dickinson, USA) were used. The cytokines/chemokines were quantified by multiplex microsphere beads according to the manufacturer's instructions. The beads of known size and fluorescence were mixed with the recombinant standards or unknown samples and incubated with the PE conjugated detection antibodies to form sandwich complexes. The intensity of PE fluorescence of each sandwich complex indicates the concentration of analyzed entity.

Human Th1/Th2/Th17 Cytokine Kit measured concentration of following cytokines: interleukin-2 (IL-2), interleukin-4 (IL-4), interleukin-6 (IL-6), interleukin-10 (IL-10), interleukin-17A (IL-17A), interferon-γ (IFN-γ) and tumor necrosis factor α (TNF). The standard curve for each cytokine covered a defined set of concentrations from 20 to 5000 pg/mL. Human Chemokine Kit quantitatively measured interleukin-8 (CXCL8/IL-8), RANTES (CCL5/RANTES), monokine induced by interferon-γ (CXCL9/MIG), monocyte chemoattractant protein-1 (CCL2/MCP-1) and interferon-γ induced protein-10 (CXCL10/IP-10) levels in a single sample. The standard curve for each chemokine covered a defined set of concentrations from 10 to 2500 pg/mL. Serum samples were diluted 1:2 prior to procedure to ensure that their median fluorescence values fall within the range of the generated chemokine standard curve. The sample acquisition was performed with use of FACSCanto II flow cytometer (Becton Dickinson, USA) and data were analyzed using FCAPArray™ software.

### Statistical analysis

Statistical analysis was performed with STATISTICA 12.0 software for Windows. The results were shown as mean values with standard deviation or median. Wilcoxon matched-pairs signed rank test was used due to nonparametric data distribution. A value of p < 0.05 was considered to be statistically significant.

## References

[1] Caligaris-Cappio F, Hamblin TJ (1999). B-cell chronic lymphocytic leukemia: a bird of a different feather. J Clin Oncol.

[2] Chiorazzi N, Rai KR, Ferrarini M (2005). Chronic lymphocytic leukemia. N Engl J Med.

[3] Sagatys EM, Zhang L (2012). Clinical and laboratory prognostic indicators in chronic lymphocytic leukemia. Cancer Control.

[4] Stilgenbauer S (2006). Chromic lymphocytic leukemia: genetics for predicting outcome. Hematology.

[5] Crespo M, Bosch F, Villamor N, Bellosillo B, Colomer D, Rozman M, Marcé S, López-Guillermo A, Campo E, Montserrat E (2003). ZAP-70 expression as a surrogate for immunoglobulin-variable-region mutations in chronic lymphocytic leukemia. N Engl J Med.

[6] Cramer P, Hallek M, Eichhorst B (2016). State-of-the-Art Treatment and Novel Agents in Chronic Lymphocytic Leukemia. Oncol Res Treat.

[7] Döhner H, Stilgenbauer S, Benner A, Leupolt E, Kröber A, Bullinger L, Döhner K, Bentz M, Lichter P (2000). Genomic aberrations and survival in chronic lymphocytic leukemia. N Engl J Med.

[8] Malcikova J, Smardova J, Rocnova L, Tichy B, Kuglik P, Vranova V, Cejkova S, Svitakova M, Skuhrova Francova H, Brychtova Y, Doubek M, Brejcha M, Klabusay M (2009). Monoallelic and biallelic inactivation of TP53 gene in chronic lymphocytic leukemia: selection, impact on survival, and response to DNA damage. Blood.

[9] Zenz T, Benner A, Döhner H, Stilgenbauer S (2008). Chronic lymphocytic leukemia and treatment resistance in cancer: the role of the p53 pathway. Cell Cycle.

[10] Routledge DJ, Bloor AJ (2016). Recent advances in therapy of chronic lymphocytic leukaemia. Br J Haematol.

[11] Eichhorst B, Cramer P, Hallek M (2016). Initial therapy of chronic lymphocytic leukemia. Semin Oncol.

[12] Burger JA, Chiorazzi N (2013). B cell receptor signaling in chronic lymphocytic leukemia. Trends Immunol.

[13] Han TT, Fan L, Li JY, Xu W (2014). Role of chemokines and their receptors in chronic lymphocytic leukemia: function in microenvironment and targeted therapy. Cancer Biol Ther.

[14] Burger JA (2012). Targeting the microenvironment in chronic lymphocytic leukemia is changing the therapeutic landscape. Curr Opin Oncol.

[15] Efremov DG, Gobessi S, Longo PG (2007). Signaling pathways activated by antigen-receptor engagement in chronic lymphocytic leukemia B-cells. Autoimmun Rev.

[16] Davids MS, Brown JR (2012). Targeting the B cell receptor pathway in chronic lymphocytic leukemia. Leuk Lymphoma.

[17] Honigberg LA, Smith AM, Sirisawad M, Verner E, Loury D, Chang B, Li S, Pan Z, Thamm DH, Miller RA, Buggy JJ (2010). The Bruton tyrosine kinase inhibitor PCI-32765 blocks B-cell activation and is efficacious in models of autoimmune disease and B-cell malignancy. Proc Natl Acad Sci USA.

[18] Herman SE, Gordon AL, Hertlein E, Ramanunni A, Zhang X, Jaglowski S, Flynn J, Jones J, Blum KA, Buggy JJ, Hamdy A, Johnson AJ, Byrd JC (2011). Bruton tyrosine kinase represents a promising therapeutic target for treatment of chronic lymphocytic leukemia and is effectively targeted by PCI-32765. Blood.

[19] Ponader S, Chen SS, Buggy JJ, Balakrishnan K, Gandhi V, Wierda WG, Keating MJ, O’Brien S, Chiorazzi N, Burger JA (2012). The Bruton tyrosine kinase inhibitor PCI-32765 thwarts chronic lymphocytic leukemia cell survival and tissue homing *in vitro* and *in vivo*. Blood.

[20] Byrd JC, Furman RR, Coutre SE, Burger JA, Blum KA, Coleman M, Wierda WG, Jones JA, Zhao W, Heerema NA, Johnson AJ, Shaw Y, Bilotti E (2015). Three-year follow-up of treatment-naïve and previously treated patients with CLL and SLL receiving single-agent ibrutinib. Blood.

[21] Byrd JC, Furman RR, Coutre SE, Flinn IW, Burger JA, Blum KA, Grant B, Sharman JP, Coleman M, Wierda WG, Jones JA, Zhao W, Heerema NA (2013). Targeting BTK with ibrutinib in relapsed chronic lymphocytic leukemia. N Engl J Med.

[22] O’Brien S, Furman RR, Coutre SE, Sharman JP, Burger JA, Blum KA, Grant B, Richards DA, Coleman M, Wierda WG, Jones JA, Zhao W, Heerema NA (2014). Ibrutinib as initial therapy for elderly patients with chronic lymphocytic leukaemia or small lymphocytic lymphoma: an open-label, multicentre, phase 1b/2 trial. Lancet Oncol.

[23] Byrd JC, Brown JR, O’Brien S, Barrientos JC, Kay NE, Reddy NM, Coutre S, Tam CS, Mulligan SP, Jaeger U, Devereux S, Barr PM, Furman RR, RESONATE Investigators (2014). Ibrutinib versus ofatumumab in previously treated chronic lymphoid leukemia. N Engl J Med.

[24] Hillmen P (2011). Using the biology of chronic lymphocytic leukemia to choose treatment. Hematology Am Soc Hematol Educ Program.

[25] Herishanu Y, Katz BZ, Lipsky A, Wiestner A (2013). Biology of chronic lymphocytic leukemia in different microenvironments: clinical and therapeutic implications. Hematol Oncol Clin North Am.

[26] Niemann CU, Herman SE, Maric I, Gomez-Rodriguez J, Biancotto A, Chang BY, Martyr S, Stetler-Stevenson M, Yuan CM, Calvo KR, Braylan RC, Valdez J, Lee YS (2016). Disruption of *in vivo* Chronic Lymphocytic Leukemia Tumor-Microenvironment Interactions by Ibrutinib—Findings from an Investigator-Initiated Phase II Study. Clin Cancer Res.

[27] Farooqui MZ, Valdez J, Martyr S, Aue G, Saba N, Niemann CU, Herman SE, Tian X, Marti G, Soto S, Hughes TE, Jones J, Lipsky A (2015). Ibrutinib for previously untreated and relapsed or refractory chronic lymphocytic leukaemia with TP53 aberrations: a phase 2, single-arm trial. Lancet Oncol.

[28] O’Garra A, Vieira P (2004). Regulatory T cells and mechanisms of immune system control. Nat Med.

[29] Giannopoulos K, Schmitt M, Kowal M, Wlasiuk P, Bojarska-Junak A, Chen J, Rolinski J, Dmoszynska A (2008). Characterization of regulatory T cells in patients with B-cell chronic lymphocytic leukemia. Oncol Rep.

[30] Giannopoulos K, Dmoszynska A, Kowal M, Wasik-Szczepanek E, Bojarska-Junak A, Rolinski J, Döhner H, Stilgenbauer S, Bullinger L (2009). Thalidomide exerts distinct molecular antileukemic effects and combined thalidomide/fludarabine therapy is clinically effective in high-risk chronic lymphocytic leukemia. Leukemia.

[31] Beyer M, Kochanek M, Darabi K, Popov A, Jensen M, Endl E, Knolle PA, Thomas RK, von Bergwelt-Baildon M, Debey S, Hallek M, Schultze JL (2005). Reduced frequencies and suppressive function of CD4+CD25hi regulatory T cells in patients with chronic lymphocytic leukemia after therapy with fludarabine. Blood.

[32] Dubovsky JA, Beckwith KA, Natarajan G, Woyach JA, Jaglowski S, Zhong Y, Hessler JD, Liu TM, Chang BY, Larkin KM, Stefanovski MR, Chappell DL, Frissora FW (2013). Ibrutinib is an irreversible molecular inhibitor of ITK driving a Th1-selective pressure in T lymphocytes. Blood.

[33] Bernard S, Danglade D, Gardano L, Laguillier C, Lazarian G, Roger C, Thieblemont C, Marzec J, Gribben J, Cymbalista F, Varin-Blank N, Ledoux D, Baran-Marszak F (2015). Inhibitors of BCR signalling interrupt the survival signal mediated by the micro-environment in mantle cell lymphoma. Int J Cancer.

